# Smart Vesicle Therapeutics: Engineering Precision at the Nanoscale

**DOI:** 10.3390/pharmaceutics17121588

**Published:** 2025-12-09

**Authors:** Luciano A. Benedini, Paula V. Messina

**Affiliations:** 1 INQUISUR—CONICET, Department of Chemistry, Universidad Nacional Del Sur, Bahía Blanca B8000CPB, Argentina; lbenedini@uns.edu.ar; 2 Department of Biology, Biochemistry and Pharmacy, Universidad Nacional Del Sur, Bahía Blanca B8000CPB, Argentina

**Keywords:** liposomes, exosomes, extracellular vesicles, niosomes, polymersomes, active and passive targeting, pharmacokinetics, isolation/synthesis, functionalization, modified release

## Abstract

Smart vesicle therapeutics represent a transformative frontier in nanomedicine, offering precise, biocompatible, and adaptable platforms for drug delivery and theranostic applications. This review explores recent advances in the design and engineering of liposomes, niosomes, polymersomes, and extracellular vesicles (EVs), emphasizing their capacity to integrate therapeutic and diagnostic functions within a single nanoscale system. By tailoring vesicle size, composition, and surface chemistry, researchers have achieved improved pharmacokinetics, reduced immunogenicity, and fine-tuned control of drug release. Stimuli-responsive vesicles activated by pH, temperature, and redox gradients, or external fields enable spatiotemporal regulation of therapeutic action, while hybrid bio-inspired systems merge synthetic stability with natural targeting and biocompatibility. Theranostic vesicles further enhance precision medicine by allowing real-time imaging, monitoring, and adaptive control of treatment efficacy. Despite these advances, challenges in large-scale production, reproducibility, and regulatory standardization still limit clinical translation. Emerging solutions—such as microfluidic manufacturing, artificial intelligence-guided optimization, and multimodal imaging integration—are accelerating the development of personalized, high-performance vesicular therapeutics. Altogether, smart vesicle platforms exemplify the convergence of nanotechnology, biotechnology, and clinical science, driving the next generation of precision therapies that are safer, more effective, and tailored to individual patient needs.

## 1. Introduction

**Figure 1:**
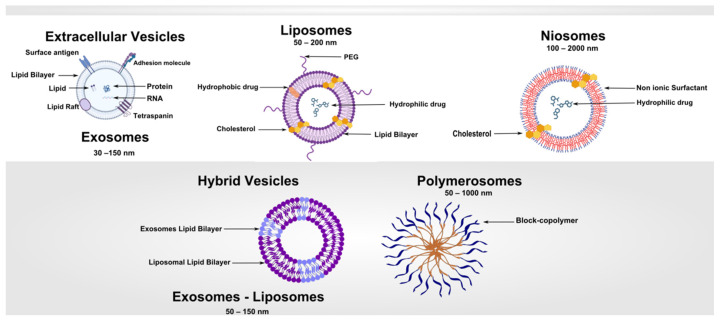
Vesicle-Based Systems: Schematic overview of the main classes of vesicle-derived and vesicle-inspired nanocarriers, highlighting their structural features, composition, and functional principles.

Vesicle-based systems have emerged as one of the most versatile and clinically impactful platforms in modern nanomedicine [1]. These systems, ranging from traditional liposomes to advanced polymersomes [2,3] and extracellular vesicles [4,5], provide a structural framework that can encapsulate and protect therapeutic agents, enhance bioavailability, and enable targeted delivery to specific tissues or cellular compartments (Figure 1). The journey from early liposomal constructs in the 1960s to clinically approved nanoformulations exemplifies a confluence of interdisciplinary research, integrating materials science, pharmacology, cell biology, and clinical medicine [6]. Liposomes are the most established carriers used in clinics, but their limited stability restricts their use in long-term antimicrobial treatments [7]. Polymersomes are more durable, but they come with increased complexity and possible toxicity [8]. Exosomes provide distinct benefits for regeneration and immune response, making them the best option for bone healing, although their variability is a significant challenge [9,10,11]. Hybrid systems offer a sensible way to combine compatibility with stability, yet reproducibility issues continue [12]. Lastly, metal–organic framework (MOF)-based vesicles have exceptional capacity for loading and controlled release, but worries about metal toxicity hinder their immediate application [13,14]. The clinical motivation for vesicular systems lies primarily in addressing the limitations of conventional therapeutics, including poor solubility, rapid systemic clearance, off-target toxicity, and immunogenicity. Liposomes, the first clinically approved vesicles, demonstrated how encapsulation could reduce cardiotoxicity of doxorubicin while maintaining antitumor efficacy, providing proof-of-concept for the broader field [15]. The subsequent exploration of non-ionic surfactant vesicles (niosomes) [16], block-copolymer vesicles (polymersomes), and naturally derived extracellular vesicles expanded the therapeutic landscape, introducing opportunities for programmable release, active targeting, and theranostic integration [17]. Contemporary research is increasingly focused on enhancing specificity through surface functionalization, controlled pharmacokinetics, and incorporation of stimuli-responsive mechanisms. The integration of extracellular vesicles, particularly exosomes, leverages innate biological targeting and intercellular communication pathways, presenting a paradigm shift in how drugs can be delivered with minimal immunogenicity [18,19]. As the field moves toward personalized medicine, vesicle-based systems are expected to play a pivotal role in tailoring therapies to patient-specific pathophysiology. Key drivers shaping the evolution of vesicular drug carriers include advances in nano-fabrication techniques, computational modeling for design optimization, and a growing understanding of the nano–bio interface. However, challenges remain: standardization of isolation methods, scalable manufacturing, immunological safety, and clinical translation of complex vesicles such as EVs. This review synthesizes the state-of-the-art knowledge on vesicle-based drug delivery, encompassing the physicochemical principles, vesicle classes, methods of synthesis and isolation, functionalization strategies, pharmacokinetics, targeting approaches, clinical applications, and regulatory considerations.

## 2. Fundamentals of Vesicle-Based Systems

Vesicles are nanoscale or microscale spherical structures enclosed by one or more bilayers, capable of encapsulating hydrophilic drugs within the aqueous core and hydrophobic compounds within the lipidic membrane. The defining characteristic of vesicular systems is their amphiphilic architecture, which provides both structural stability and the capacity to interact with biological membranes [20].

### 2.1. Vesicle Morphology and Stability

The morphology of vesicles—including size, lamellarity, and membrane composition—profoundly influences their pharmacokinetics, biodistribution, and cellular uptake. Small unilamellar vesicles (SUVs), typically 20–100 nm in diameter, are often employed for intravenous applications due to their ability to evade rapid clearance and penetrate tissues efficiently. Large unilamellar vesicles (LUVs) and multilamellar vesicles (MLVs) are used in applications requiring higher encapsulation capacity, slow release, or depot formulations [21]. The stability of vesicles is influenced by lipid phase behavior, membrane rigidity, and the presence of stabilizing agents such as cholesterol, which modulates bilayer packing and reduces permeability [22,23]. In polymersomes, amphiphilic block copolymers form thicker, more mechanically robust membranes than lipid bilayers, allowing for greater stability in circulation and resistance to enzymatic degradation [8]. Similarly, niosomes constructed from non-ionic surfactants offer chemical stability and reduced susceptibility to oxidation, making them suitable for storage and industrial-scale applications [16,24]. Table 1 summarizes the key structural, functional, and performance-related differences among the principal vesicle types.

### 2.2. Drug Encapsulation and Release Mechanisms

Encapsulation efficiency in vesicular systems is governed by a combination of factors, including vesicle composition, size, preparation method, and the physicochemical nature of the therapeutic cargo. Hydrophilic drugs preferentially accumulate in the aqueous core, whereas hydrophobic or amphiphilic molecules partition into the lipid bilayer or polymeric membrane [38]. This structural versatility allows vesicles to simultaneously accommodate molecules with diverse solubility profiles, offering a major advantage over conventional delivery systems. Moreover, the ability to fine-tune encapsulation parameters provides a means to optimize drug loading, stability, and release characteristics according to the intended therapeutic application [27].

Drug release from vesicular carriers can be achieved through both passive and stimuli-responsive mechanisms, each providing distinct therapeutic advantages [39]. Passive release typically occurs via gradual diffusion of the encapsulated drug across the vesicle bilayer or through natural degradation of the carrier in biological fluids. This mechanism ensures sustained therapeutic levels over extended periods, making it particularly valuable for chronic conditions requiring long-term dosing. For example, liposomal formulations of amphotericin B exploit slow release to reduce systemic toxicity while maintaining antifungal efficacy [40].

In contrast, stimuli-responsive systems introduce a higher level of spatiotemporal control, leveraging environmental or externally applied cues to trigger drug release at the desired site of action. Internal stimuli such as pH, redox potential, and enzymatic activity are frequently exploited. pH-sensitive liposomes, for instance, destabilize in acidic environments characteristic of tumor tissues or endosomal compartments, releasing chemotherapeutic drugs specifically at the pathological site. A clinically relevant example is liposomal doxorubicin (Doxil^®^/Caelyx^®^), which benefits from prolonged circulation and preferential accumulation in tumors via the enhanced permeability and retention (EPR) effect, while its release is facilitated within the acidic tumor microenvironment [41]. Similarly, redox-sensitive polymersomes are engineered to disassemble in the presence of elevated intracellular glutathione concentrations, thereby promoting cytosolic drug delivery in cancer cells [42]. External triggers such as temperature, light, magnetic fields, or ultrasound provide further versatility. Thermosensitive liposomes (e.g., ThermoDox^®^) exemplify this approach by releasing doxorubicin upon localized heating of tumor tissues, enabling precise, site-specific chemotherapy [42,43].

Beyond release mechanisms, the physicochemical properties of vesicles critically determine pharmacokinetics and biodistribution [44]. Vesicle size strongly influences circulation half-life and tissue penetration, with nanoscale vesicles (<200 nm) exhibiting improved tumor accumulation through the EPR effect, whereas larger vesicles are more readily sequestered by the mononuclear phagocyte system. Surface charge also plays a pivotal role: positively charged vesicles interact more efficiently with negatively charged cellular membranes, enhancing uptake but leading to rapid clearance by the reticuloendothelial system (RES). In contrast, neutral or slightly negative vesicles generally display prolonged circulation times, reduced immunogenicity, and improved systemic stability. Surface modifications such as polyethylene glycol (PEGylation) further extend circulation half-life by reducing protein adsorption and opsonization, granting vesicles “stealth” properties [45].

Altogether, the interplay between vesicle architecture, encapsulation strategy, and release mechanism enables the rational design of drug delivery systems that balance stability in circulation with efficient and targeted drug release. By integrating controlled release technologies with clinically validated strategies, vesicular systems such as Doxil^®^ [15,41] and ThermoDox^®^ [46] highlight the translational potential of this platform, demonstrating how nanoscale engineering can reshape therapeutic outcomes in oncology, infectious diseases, and beyond.

### 2.3. Design Considerations and the Nano–Bio Interface

Understanding the nano–bio interface is fundamental for the rational design of vesicular carriers that achieve their intended therapeutic effects [47]. Once introduced into biological fluids, vesicles rapidly adsorb proteins, forming a dynamic “protein corona” that modulates immune recognition, circulation half-life, and biodistribution [48,49].

**Figure 2:**
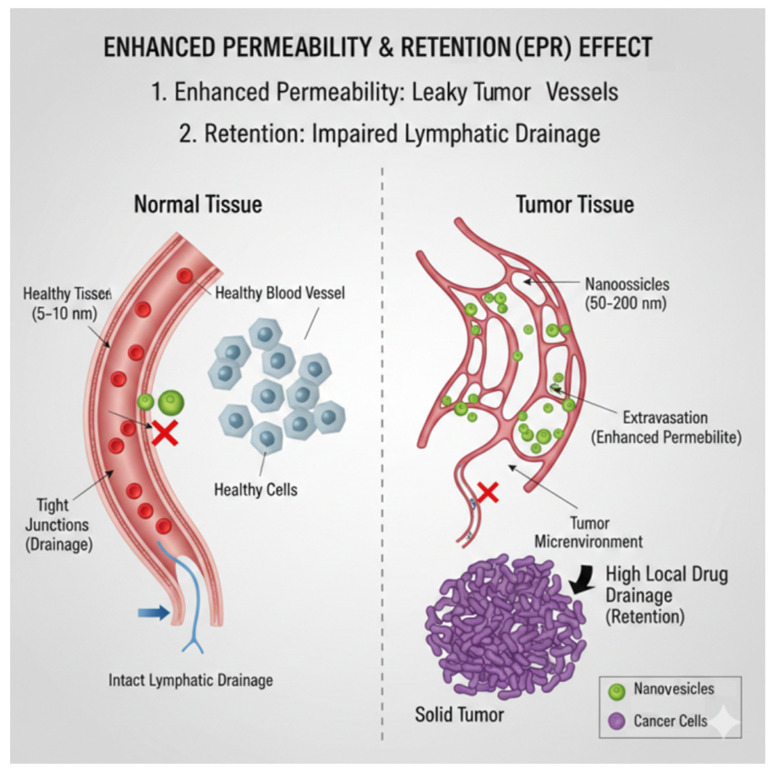
The EPR effect is a key mechanism used in nanomedicine for targeting solid tumors passively. The figure shows the differences between blood vessels in healthy tissue (**Left**) and tumor tissue (**Right**). Normal Tissue (**Left**): Healthy blood vessels have tight junctions between endothelial cells and a working lymphatic drainage system. Nanovesicles, which are typically 50 to 200 nm in size, cannot leak into the tissue because of these tight junctions and are cleared efficiently by the lymphatic system. Tumor Tissue (**Right**): The rapidly growing tumor creates abnormal blood vessels (neovasculature) that have large gaps, usually 100 to 200 nm wide. Improved Permeability: These leaky vessels allow nanovesicles that carry drugs to easily leak into the tumor microenvironment. Retention: Solid tumors often have a poor or damaged lymphatic drainage system, indicated by the blocked vessel. This means that once nanovesicles build up in the tumor area, they stay there for a long time, leading to a very high local concentration of drugs. This combined effect allows nanovesicles to build up selectively in the tumor mass, maximizing treatment effectiveness while reducing harm to healthy tissues.

Strategies such as PEGylation can reduce protein adsorption, extend systemic circulation, and enhance passive targeting via the enhanced permeability and retention (EPR) effect (Figure 2) [50]. Clinically, PEGylated liposomal formulations such as Doxil^®^/Caelyx^®^ exemplify this principle, achieving prolonged circulation, reduced cardiotoxicity, and improved tumor accumulation compared to free doxorubicin [41]. Although the EPR effect is a key idea in nanomedicine, its application in clinics has been inconsistent. Evidence shows that EPR intensity varies a lot across tumor types, stages of disease, and importantly, between patients [51]. This variability is affected by several clinical factors, such as abnormal or insufficient tumor blood supply, high interstitial fluid pressure, inconsistent blood flow, and the presence of dense stromal barriers. As a result, many nanocarrier systems, even though they show strong passive accumulation in mouse tumor models, have limited or uneven uptake in humans. These challenges show the need to treat the EPR effect as a context-dependent mechanism of tumor targeting rather than a universal one. Several strategies that recognize the limits of depending solely on the EPR effect have been suggested to address weak or variable EPR profiles [52,53,54]:

- Active targeting: Adding antibodies, peptides, aptamers, or small molecules to improve how cells recognize and take in the treatment, regardless of blood vessel permeability.
- Vascular modulation: Using vasodilators, anti-fibrotic agents, or normalization strategies to temporarily increase blood flow and help nanoparticles exit blood vessels.
- Externally triggered delivery: Using ultrasound, heat, magnetic fields, or light to locally increase permeability or trigger the release of treatment when needed.
- Tumor microenvironment remodeling: Lowering interstitial fluid pressure or reducing the density of the extracellular matrix to allow deeper penetration of nanoparticles.
- Patient stratification approaches: Using pre-treatment imaging or functional biomarkers to find individuals with favorable EPR profiles and tailor nanotherapeutic interventions for them.

In addition to the limitations of the EPR effect, several safety and reproducibility issues deserve careful attention. For instance, PEG immunogenicity, which is increasingly documented through the development of anti-PEG antibodies, may compromise circulation times, change pharmacokinetics, and, in sensitized patients, even cause hypersensitivity reactions [55].

In parallel, naturally occurring extracellular vesicles (EVs) provide unique inspiration for therapeutic delivery [56]. Secreted by cells, EVs carry intrinsic targeting ligands, membrane proteins, and nucleic acids that enable highly specific communication with recipient cells while minimizing immune activation. This natural targeting capacity is being actively explored in regenerative medicine and gene therapy [1,4,5,44,56]. For instance, mesenchymal stem cell–derived EVs are under investigation for promoting tissue repair and bone regeneration, as they can deliver growth factors, microRNAs, and other bioactive molecules directly to injured tissues. Similarly, tumor-derived EVs, when properly engineered, are being explored as carriers for RNA-based therapeutics, highlighting the versatility and clinical potential of EV-inspired strategies [9,57]. Extracellular vesicles exhibit inherent biological variability arising from their cellular origin, isolation method, and batch-to-batch heterogeneity, which complicates standardization and regulatory approval [58].

The rational design of synthetic or biologically inspired vesicle-based carriers requires careful balancing of multiple, often competing, and parameters. High encapsulation efficiency, structural stability during storage and circulation, selective tissue or cellular targeting, efficient uptake, and precisely controlled release are all essential to therapeutic success. To meet these demands, researchers increasingly employ computational modeling, high-throughput screening and advanced imaging techniques to predict vesicle behavior in complex biological environments. These strategies guide formulation optimization prior to in vivo evaluation, reducing trial-and-error experimentation and accelerating the translation of vesicular systems into clinical practice [59,60].

Concrete examples illustrate how these design considerations converge in real applications. Thermosensitive liposomes such as ThermoDox^®^ employ local hyperthermia to trigger controlled release of doxorubicin at tumor sites, achieving spatiotemporal precision [43]. In contrast, cationic liposomes have been optimized for the delivery of genetic material, including mRNA vaccines, where their electrostatic interaction with nucleic acids enables efficient encapsulation and cellular transfection [61,62]. Meanwhile, stem-cell–derived EVs are progressing toward clinical trials in cardiovascular and musculoskeletal disorders, showing promise in accelerating bone healing [63] and cardiac repair [64].

Beyond their biological relevance, the successful clinical implementation of EVs critically depends on standardized, reproducible, and scalable production pipelines [65]. Upstream, the field has moved from conventional planar flasks toward advanced scale-up platforms such as hollow-fiber, fixed-bed, and stirred-tank bioreactors, which allow controlled physicochemical conditions, automated feeding strategies, and high-density cultures that markedly increase EV productivity [66]. Importantly, parameters such as shear stress, oxygen availability, and nutrient gradients can significantly affect EV cargo composition and functional potency, reinforcing the need for well-defined bioprocess controls. Downstream, the transition toward clinical-grade purification has highlighted the advantages of tangential flow filtration (TFF) and size-exclusion chromatography (SEC), which enable continuous processing, reduced shear, and improved removal of proteins, lipoproteins, and other non-vesicular nanoparticles [67]. Hybrid workflows that combine TFF pre-concentration with SEC polishing are now considered best practice for obtaining high-purity EV preparations while preserving vesicle integrity. Alongside these advances, regulatory bodies increasingly emphasize quantitative quality attributes, including particle-to-protein and particle-to-lipid ratios, profiling of non-vesicular contaminants, sterility and endotoxin testing, and robust markers of identity and potency. These criteria are essential for minimizing batch-to-batch variability, a well-recognized challenge derived from donor heterogeneity, cell-line drift, and process sensitivity. Finally, several EV-based products have progressed to clinical evaluation, particularly in wound healing, osteo-regeneration, immune modulation, and cancer therapy. Early-phase trials have demonstrated encouraging safety profiles and preliminary efficacy signals [68], but they also underscore the urgent need for harmonized good manufacturing practice (GMP)-compliant [69], well-defined release specifications, and cross-study comparability. Recent comprehensive reviews offer thoughtful analyses of current bioprocessing technologies, regulatory expectations, and the evolving clinical landscape and are recommended for readers seeking deeper insight into these emerging standards [70].

Altogether, the integration of knowledge on the nano–bio interface, coupled with advances in vesicle design and characterization, is reshaping how therapeutic cargos are delivered. By combining strategies such as PEGylation, stimuli-responsive release, and biologically inspired EV engineering, researchers are developing next-generation carriers capable of balancing circulation stability with targeted, efficient, and safe drug delivery across oncology, regenerative medicine, and gene therapy. However, the long-term safety of many vesicle-based platforms remains insufficiently characterized, particularly regarding chronic exposure, potential accumulation, and immune modulation. Addressing these aspects is essential to ensure the reproducibility, safety, and clinical reliability of next-generation vesicular nanomedicines.

## 3. Liposomes: Design, Preparation, and Clinical Applications

Liposomes represent the most extensively studied vesicular drug delivery systems and were the first to achieve widespread clinical application. These spherical vesicles, composed primarily of phospholipid bilayers, can encapsulate hydrophilic drugs in their aqueous core and hydrophobic compounds within the lipid membrane. Their versatility arises from the ability to modulate key parameters such as size, lamellarity, lipid composition, and surface functionality. Conventional liposomes are typically constructed from naturally derived phosphatidylcholine, phosphatidylethanolamine, and cholesterol, whereas synthetic analogs provide enhanced stability and allow fine-tuning of pharmacokinetics for specific therapeutic purposes [26,71].

Over time, liposome preparation techniques have evolved from basic laboratory-scale protocols to sophisticated methods enabling reproducibility and scalability. The classical thin-film hydration method involves dissolving lipids in organic solvents, forming a dry lipid film, and subsequently hydrating it with an aqueous buffer to yield multilamellar vesicles (MLVs) [72]. These MLVs can then be downsized to large unilamellar vesicles (LUVs) [73] or small unilamellar vesicles (SUVs) through extrusion, sonication, or high-pressure homogenization. Alternative methods such as ethanol injection and reverse-phase evaporation offer additional control over vesicle size and encapsulation efficiency. More recently, microfluidic hydrodynamic focusing has emerged as a powerful approach, enabling the generation of monodisperse liposomes with narrow size distributions suitable for large-scale production [74]. Complementary to these advances, remote loading techniques, most notably pH and ammonium sulfate gradient methods, permit high drug-to-lipid ratios and efficient entrapment of ionizable drugs [75]. A prominent example of this strategy is the PEGylated liposomal doxorubicin formulation, Doxil^®^/Caelyx^®^, which has become a landmark product in nanomedicine [41].

The functional diversity of liposomes is reflected in their various subtypes. Conventional liposomes represent the basic platform, whereas stealth liposomes employ polyethylene glycol (PEG) chains on their surface to extend circulation half-life by sterically hindering opsonin adsorption and reducing clearance by the mononuclear phagocyte system (MPS) [45]. pH-sensitive liposomes are designed to destabilize in acidic environments such as tumor microenvironments or endosomal compartments, facilitating site-specific drug release [25]. Cationic liposomes, on the other hand, are engineered for nucleic acid delivery due to their ability to form electrostatic complexes with negatively charged oligonucleotides, though their use is often limited by potential immunogenicity [76]. Finally, ligand-targeted liposomes incorporate antibodies, peptides, or small molecules on their surface to enhance specificity toward diseased tissues or cell populations [77].

The clinical translation of liposomal systems highlights their impact across multiple therapeutic domains. In oncology, Doxil^®^/Caelyx^®^ (PEGylated liposomal doxorubicin) exemplifies how lipid-based encapsulation can reduce cardiotoxicity, extend half-life, and improve tumor targeting [41]. In infectious diseases, AmBisome^®^ (liposomal amphotericin B) significantly enhances antifungal efficacy while minimizing nephrotoxicity, representing a major advancement over free drug formulations [78]. More recently, Onivyde^®^ (liposomal irinotecan) has demonstrated improved tolerability and efficacy within chemotherapy regimens, further extending the reach of liposomal technology [79]. Collectively, these examples underscore how rational design, advanced preparation methods, and tailored functionalization converge to make liposomes one of the most clinically successful nanocarrier systems to date.

Looking forward, next-generation liposomal platforms are being developed to overcome current limitations and expand therapeutic horizons. Hybrid liposomes that integrate polymers or inorganic components are under investigation to enhance stability, modulate release kinetics, and introduce multifunctionality [12]. Immunoliposomes, decorated with antibodies or immune ligands, are being designed for precision oncology, enabling selective recognition of tumor-associated antigens while minimizing off-target effects [80,81]. In regenerative medicine, liposomes are increasingly studied as carriers for growth factors, osteoinductive molecules, and nucleic acids to promote bone repair and tissue regeneration, offering promising strategies for orthopedic applications. These innovations highlight the continuous evolution of liposomal systems, reinforcing their central role in the future of nanomedicine.

## 4. Niosomes: Composition, Preparation, and Applications

Niosomes are vesicular systems composed primarily of non-ionic surfactants, often supplemented with cholesterol to enhance membrane rigidity and reduce drug leakage [16,24]. Compared to liposomes, they offer improved chemical and physical stability, lower production costs, and reduced susceptibility to oxidative degradation, making them attractive alternatives for large-scale pharmaceutical applications.

The composition of niosomes typically involves spans, tweens, or other non-ionic surfactants with different hydrophilic-lipophilic balance (HLB) values, which strongly influence vesicle size, lamellarity, and encapsulation efficiency. Cholesterol incorporation is critical for modulating membrane fluidity and permeability, leading to enhanced stability and sustained release characteristics [82]. Recent research also explores the use of surfactant mixtures and the inclusion of charged lipids or polymers, aiming to further improve stability and provide opportunities for surface functionalization and targeted delivery [83,84].

Preparation techniques for niosomes mirror those employed for liposomes, including thin-film hydration, reverse-phase evaporation, and microfluidic mixing. These methods allow high encapsulation efficiency of both hydrophilic and lipophilic drugs, with process parameters carefully tuned to control vesicle size distribution and release kinetics. Advances in microfluidic technologies have enabled scalable production of monodisperse niosomes, while remote loading strategies are beginning to be adapted to improve drug-to-surfactant ratios and therapeutic performance [85].

The versatility of niosomes has been demonstrated across diverse drug delivery routes, including dermal, ocular, oral, and parenteral administration. Their structural robustness makes them compatible with integration into hydrogels, creams, and transdermal patches, expanding their potential for localized and sustained delivery. Preclinical studies have reported promising outcomes in the delivery of anticancer drugs, antivirals, and vaccines, with improvements in bioavailability, pharmacokinetics, and controlled release profiles compared to free drugs. For example, niosomal formulations of doxorubicin and cisplatin have shown enhanced tumor accumulation and reduced systemic toxicity [86], while niosomal vaccine candidates have demonstrated improved antigen stability and immunogenicity [87].

Looking ahead, niosomes are increasingly being explored as platforms for next-generation therapies. Their low-cost and scalable productions make them appealing for applications in low-resource settings, particularly for vaccines and antimicrobial therapies. In regenerative medicine, niosomes are being studied as carriers for growth factors and genetic material, with the goal of promoting tissue repair and wound healing. Furthermore, hybrid systems that combine niosomes with polymers, nanoparticles, or stimuli-responsive components hold promise for creating multifunctional carriers capable of precision targeting and controlled release. These advances position niosomes as a complementary and versatile alternative to liposomes, with growing potential to contribute significantly to future nanomedicine.

## 5. Polymersomes: Design, Stimuli-Responsiveness, and Applications

Polymersomes are vesicular nanocarriers formed from amphiphilic block copolymers, distinguished from liposomes and niosomes by their thicker membranes, which confer superior mechanical robustness and tunable permeability. This structural advantage allows them to maintain integrity under shear stress and during extended circulation, making them particularly attractive for demanding therapeutic applications [2,8,42].

The design of polymersomes can be precisely tailored by adjusting polymer chain length, hydrophobic-to-hydrophilic ratios, and chemical composition. These parameters control vesicle size, membrane rigidity, and responsiveness to external or internal stimuli. As a result, polymersomes are versatile platforms capable of encapsulating a wide range of cargos, including proteins, peptides, nucleic acids, and small-molecule drugs, while preserving their activity and stability. Their modularity also permits the incorporation of functional groups for targeting, imaging, or triggered release.

A defining feature of polymersomes is their capacity for stimuli-responsive behavior. Redox-sensitive polymersomes exploit intracellular glutathione gradients to release drugs selectively within the cytosol [88], while pH-sensitive systems destabilize in acidic tumor microenvironments or endosomal compartments, enabling site-specific drug delivery [29]. Thermo-responsive and photo-responsive polymers introduce further precision, with the latter enabling spatiotemporal control over cargo release through light activation [89]. Such systems open the possibility of on-demand therapy, combining drug delivery with external control for enhanced safety and efficacy.

Preclinical studies have demonstrated the promise of polymersomes in diverse applications, ranging from anticancer therapy and gene delivery to immunotherapy and vaccination [28]. For example, polymersomes loaded with siRNA or CRISPR components have achieved efficient intracellular delivery and gene silencing in tumor models [90], while immune-stimulating polymersomes are under investigation for enhancing antigen presentation in cancer vaccines [91]. Although clinical translation remains in its early stages, ongoing advances in scalable synthesis, biocompatible polymer selection, and regulatory acceptance are paving the way for their integration into human therapies.

Looking forward, polymersomes represent one of the most versatile vesicular systems under development. Their unique combination of mechanical strength, multifunctionality, and responsiveness positions them as next-generation nanocarriers for precision medicine. Beyond oncology, they hold particular promise in regenerative medicine, where controlled delivery of growth factors, nucleic acids, or signaling molecules could accelerate tissue repair and bone regeneration [30]. As polymer chemistry and nanofabrication technologies continue to evolve, polymersomes are poised to bridge the gap between preclinical innovation and clinical application, offering a highly adaptable platform for future nanotherapeutics [92].

## 6. Extracellular Vesicles and Exosomes: Biology, Isolation, and Therapeutic Potential

Extracellular vesicles (EVs), including exosomes and microvesicles, are naturally secreted nanoscale carriers composed of lipids, proteins, and nucleic acids that play a central role in intercellular communication. Their inherent biocompatibility and ability to transfer functional biomolecules have positioned them as promising candidates for therapeutic applications, bridging natural biological processes with engineered drug delivery strategies [1,4,44,56,60].

Exosomes, typically 30–150 nm in diameter, originate from endosomal multivesicular bodies that fuse with the plasma membrane to release their vesicular contents [32]. Microvesicles, ranging from 100 to 1000 nm, bud directly from the plasma membrane [93]. Due to their overlapping size, molecular cargo, and biogenesis pathways, the umbrella term “extracellular vesicles” is often preferred in scientific and clinical contexts.

Isolation and purification of EVs remain key challenges in translational research. Common strategies include ultracentrifugation, density gradient separation, size-exclusion chromatography, tangential flow filtration, and immunoaffinity capture. Each method offers distinct advantages in terms of purity, yield, and scalability, but no single technique achieves all three simultaneously. To enhance reproducibility and comparability across studies, standardized protocols such as those outlined in the Minimal Information for Studies of Extracellular Vesicles (MISEV) guidelines have been widely adopted [11].

Functionally, EVs exhibit unique advantages as natural delivery systems. Their intrinsic surface ligands enable selective targeting of recipient cells, while their low immunogenicity and capacity to cross biological barriers, including the blood–brain barrier, and broaden their therapeutic applicability. Engineered EVs can be tailored to deliver small molecules, proteins, and nucleic acids with enhanced precision, offering new possibilities for disease-specific interventions. Preclinical studies have demonstrated their potential in oncology [94], where tumor-derived EVs can be engineered for drug or RNA delivery [95]; in regenerative medicine, where mesenchymal stem cell–derived EVs accelerate tissue repair and bone regeneration [96]; and in vaccine development, where EVs act as immunogenic platforms to present antigens and stimulate robust immune responses [5,97].

Despite their promise, significant challenges must be addressed before EVs achieve widespread clinical translation. Scalable manufacturing remains difficult due to complex isolation procedures, while cargo loading efficiency and batch-to-batch reproducibility continue to limit therapeutic consistency. Furthermore, the lack of standardized potency assays and evolving regulatory frameworks adds layers of complexity to their development as approved therapeutics. Ongoing advances in bioengineering, microfluidics, and synthetic biology are beginning to address these hurdles, pointing toward a future where EV-based systems may join or complement liposomes, niosomes, and polymersomes as clinically validated nanocarriers [98].

## 7. Hybrid and Specialized Vesicles

Hybrid vesicles represent an emerging class of nanocarriers that integrate natural and synthetic components to harness complementary advantages while overcoming individual system limitations [99]. By merging features such as the biocompatibility and innate targeting properties of extracellular vesicles (EVs) with the scalability and tunable physicochemical attributes of synthetic nanocarriers, hybrid systems offer a promising avenue for next-generation drug delivery platforms.

Liposome–EV hybrids combine the structural stability and high drug-loading capacity of liposomes with the membrane proteins and targeting ligands naturally embedded in EVs [100]. This fusion enhances circulation time, immune evasion, and cell-specific uptake. Polymersome–lipid vesicles exploit the mechanical robustness and stimuli-responsiveness of block copolymers while retaining the fluidity and biocompatibility of lipid bilayers, yielding constructs with superior stability under physiological stress and precise release control [31]. Similarly, niosome–lipid conjugates incorporate low-cost, oxidation-resistant surfactants into lipidic bilayers, improving formulation stability while maintaining encapsulation versatility for both hydrophilic and hydrophobic drugs [101,102].

Beyond binary hybrids, more specialized vesicular systems are being engineered. For example, magnetoliposomes embed superparamagnetic iron oxide nanoparticles within lipid bilayers to enable externally triggered drug release and magnetic resonance imaging (MRI) contrast enhancement [103]. Immunoliposomes, which combine liposomal vesicles with monoclonal antibodies or antibody fragments, enable more selective targeting and delivery to diseased tissues such as tumors or inflamed endothelium [80,81,104]. Stimuli-responsive hybrids, integrating polymers, peptides, or inorganic nanoparticles, allow controlled drug release in response to pH, temperature, enzymatic activity, or external fields (e.g., light, ultrasound, or magnetic fields) [25,28,39,105,106].

Recent preclinical studies underscore the remarkable translational potential of hybrid vesicular systems that integrate the structural advantages of liposomes with the biological functionality of extracellular vesicles (EVs). Liposome–EV hybrids have exhibited superior efficiency in the delivery of chemotherapeutic agents to multidrug-resistant tumors, enhancing intracellular accumulation and minimizing off-target toxicity [35,36]. Similarly, magnetoliposomes and polymer–lipid nanoconstructs are being actively investigated as theranostic platforms, capable of simultaneously performing imaging and controlled drug release in the treatment of oncologic and cardiovascular disorders [107].

In the field of regenerative medicine, EV–liposome hybrid systems loaded with growth factors, siRNA, or plasmid DNA have demonstrated significant potential to accelerate tissue regeneration and bone repair, largely by improving cellular uptake and extending the bioactivity of therapeutic molecules [100,108,109]. These findings collectively suggest that hybrid lipid—EV systems could represent a new generation of biomimetic nanocarriers bridging synthetic and biological delivery approaches. Despite these advances, significant challenges remain. Hybrid vesicle design often requires complex synthesis protocols, raising concerns regarding scalability, reproducibility, and regulatory approval. Moreover, balancing the proportion of natural versus synthetic components is critical to achieving optimal stability without compromising functionality. As biofabrication methods improve and regulatory frameworks adapt, hybrid and specialized vesicles are expected to play an increasingly important role in precision nanomedicine, offering versatile platforms for targeted therapy, immunomodulation, and theranostic integration [110]. Table 2 below provides a summary of the most widely studied vesicular systems, highlighting their structural characteristics, mechanisms of drug delivery, and main advantages and limitations.

## 8. Functionalization, Targeting, and Modified Release

Vesicle functionalization [83] and controlled release mechanisms [4,15,24,25,42,73,103] are central to enhancing therapeutic efficacy while minimizing off-target effects. Surface modification enables precise cellular targeting, evasion of immune clearance, and integration of stimuli-responsive release mechanisms [45]. Functionalization strategies vary from covalent ligand attachment to non-covalent adsorption of targeting moieties, and can be combined with physicochemical modifications of the vesicle membrane to regulate drug release kinetics. The overarching goal is to achieve both active and passive targeting, optimize pharmacokinetics, and maintain payload integrity until reaching the desired tissue or cellular compartment [111,132].

### 8.1. Surface Functionalization and Targeting Strategies

Surface functionalization of vesicles plays a pivotal role in extending circulation, evading immune surveillance, and achieving precise tissue or cellular targeting. Among the most established approaches is PEGylation, where polyethylene glycol (PEG) chains are covalently attached to the vesicle surface [86]. By creating a steric barrier, PEGylation reduces protein adsorption, minimizes recognition by the mononuclear phagocyte system, and prolongs systemic circulation. This strategy has been clinically validated with PEGylated liposomal doxorubicin (Doxil), which achieves sustained plasma drug levels while reducing cardiotoxicity compared to conventional formulations [15,41]. Optimization of PEG density, chain length, and branching is crucial, as excessive PEGylation may impair cellular uptake or interfere with active targeting. Furthermore, concerns regarding anti-PEG immune responses have prompted the exploration of alternative stealth polymers, such as poly (2-oxazoline) and zwitterionic coatings, which offer similar shielding effects with reduced immunogenicity [127,133].

Beyond passive evasion, active targeting strategies aim to direct vesicles specifically to diseased tissues by decorating their surfaces with ligands that bind receptors overexpressed on target cells. Antibody-functionalized liposomes, for example, have shown selective uptake in HER2-positive breast cancer cells, illustrating the potential of immunoliposomes in oncology [116]. Similarly, vesicles modified with RGD peptides recognize integrin receptors abundantly expressed in tumor vasculature, promoting preferential accumulation within tumor tissues [134]. Aptamer-functionalized vesicles provide another versatile platform, combining the high specificity of nucleic acid recognition with chemical stability and lower production costs compared to antibodies [135]. Collectively, these ligand-based approaches enhance the spatiotemporal precision of drug delivery, reducing off-target effects and improving therapeutic efficacy.

Extracellular vesicles (EVs), naturally equipped with membrane proteins and targeting ligands, represent a distinct opportunity for surface engineering [136]. Functionalization can be achieved endogenously by genetically modifying donor cells to express desired ligands on secreted EVs, or exogenously, by chemical conjugation or fusion with synthetic nanomaterials [137]. Leveraging intrinsic proteins such as tetraspanins (CD63, CD81) not only supports targeting but also facilitates vesicle tracking and labeling [138]. Engineered EVs have been successfully employed to deliver siRNA, mRNA, and chemotherapeutics, achieving efficient uptake by recipient cells while maintaining low immunogenicity [139]. In preclinical models, these strategies have shown potential to overcome barriers such as poor cellular penetration or rapid clearance, highlighting their relevance for future translation [140].

Together, PEGylation, ligand conjugation, and EV engineering exemplify the growing sophistication of surface modification strategies. By integrating stealth properties with active targeting capabilities, researchers are advancing vesicle-based therapeutics toward higher precision, reduced systemic toxicity, and improved clinical performance.

### 8.2. Passive Targeting

Passive targeting leverages inherent physiological differences between healthy and diseased tissues, most prominently the Enhanced Permeability and Retention (EPR) effect, which enables nanoscale vesicles to accumulate selectively in tumor microenvironments [141]. This phenomenon arises from the aberrant architecture of tumor vasculature—characterized by poorly aligned endothelial cells, wide fenestrations, and defective basement membranes—that allows nanoparticles in the size range of approximately 50–200 nm to extravasate more readily than in normal tissues. At the same time, impaired lymphatic drainage in tumors reduces clearance, leading to prolonged vesicle retention and higher local drug concentrations compared to systemic circulation.

Critical design parameters, including vesicle size, surface charge, lipid or polymer composition, and stealth modifications, strongly influence the degree of EPR-mediated accumulation. Smaller vesicles < 100 nm often penetrate deeper into the tumor interstitially, whereas larger vesicles may preferentially remain near vascularized regions. Neutral or slightly negative surface charges tend to promote prolonged circulation, minimizing rapid clearance by the mononuclear phagocyte system and thereby enhancing passive deposition.

The EPR effect has historically underpinned the clinical success of several liposomal anticancer formulations, including PEGylated liposomal doxorubicin (Doxil) [122] and liposomal daunorubicin (DaunoXome) [142], which exploit passive targeting to improve pharmacokinetics and reduce systemic toxicity. However, the effectiveness of passive targeting remains highly variable, influenced by tumor type, stage, vascular density, and interstitial fluid pressure. For instance, highly vascularized tumors with chaotic vessel networks may exhibit robust EPR-mediated accumulation, while desmoplastic or hypovascular tumors show limited vesicle uptake. Moreover, interpatient variability adds another layer of complexity, limiting the predictability of therapeutic outcomes [54,123,143].

To overcome these limitations, researchers increasingly combine passive targeting with active targeting strategies, such as ligand conjugation to tumor-specific receptors, or with stimuli-responsive vesicles that release their cargo in response to local cues such as acidic pH, enzymatic activity, or external triggers like heat and ultrasound. These combinatorial approaches aim to maximize drug accumulation at diseased sites while ensuring efficient intracellular delivery. Additionally, advanced imaging modalities, including MRI and near-infrared fluorescence imaging, are being employed to monitor vesicle biodistribution in real time, allowing more personalized treatment regimens based on patient-specific tumor physiology [53,54,125,144].

Altogether, while passive targeting through the EPR effect remains a foundational principle of nanomedicine, its integration with more selective and adaptable strategies is essential to achieving consistent clinical efficacy and expanding the therapeutic reach of vesicle-based drug delivery systems.

### 8.3. Stimuli-Responsive and Modified Release Vesicles

Stimuli-responsive vesicular systems depend on design principles that let them sense and react to changes in their environment. Responsively means that a material can undergo predictable chemical or physical changes in response to internal or external stimuli like pH, temperature, redox potential, light, magnetic fields, or specific biomolecules. In polymeric and supramolecular assemblies, these responses come from functional groups or structural features that are sensitive to these triggers. Several comprehensive works covering these molecular mechanisms and their application in responsive polymeric and vesicular systems can be found in the following references [145,146,147]. A key factor in responsively is the balance between hydrophobic and hydrophilic properties of the polymer or amphiphile. Environmental changes that affect the ionization state of acidic or basic groups can shift this balance, causing variations in solubility, changes in interfacial tension, or disruption of vesicle packing. For example, protonation or deprotonation of ionizable groups can lead to swelling, collapse, or disassembly of the self-assembled structure. Hydrogen bonding and other weak, reversible interactions also play a vital role in environmental sensitivity. Changes in temperature or pH can weaken or strengthen these interactions, resulting in variations in membrane rigidity, vesicle permeability, or overall stability. Redox-sensitive groups, especially disulfide linkages, provide another way to be responsive. In reductive environments like the intracellular space rich in glutathione, disulfide bonds break, causing vesicle destabilization or polymer degradation and allowing controlled release of encapsulated agents [146]. Light-responsive vesicles use photoactive chromophores such as azobenzenes, spiropyrans, or o-nitrobenzyl groups [148]. Photoisomerization or photochemical cleavage of these groups causes changes in molecular geometry or polarity, altering bilayer packing and enabling light-triggered release. Finally, thermo-responsive materials, including polymers that exhibit lower critical solution temperature (LCST) or upper critical solution temperature (UCST) behavior, undergo coil-to-globule transitions or changes in solubility when heated or cooled [149]. These transitions can impact vesicle integrity, fluidity, or permeability. Together, these molecular mechanisms offer a flexible toolkit for designing vesicles with targeted and stimuli-dependent behaviors. By incorporating ionizable groups, reversible bonds, photoactive cores, or redox-sensitive linkages into the membrane-forming components, it becomes possible to create next-generation vesicular systems with finely tunable and predictable responsive properties.

Stimuli-responsive vesicles are engineered to release their therapeutic cargo in response to specific physiological or externally applied triggers, offering enhanced precision and spatiotemporal control compared to conventional delivery systems [25,105]. These platforms exploit differences between healthy and diseased tissues or harness controllable external cues to achieve on-demand drug release while minimizing systemic toxicity. One of the most widely studied approaches is the design of pH-sensitive vesicles, which leverage the acidic environments characteristic of tumor tissues, endosomes, and lysosomes [150]. Incorporating acid-labile lipids, such as phosphatidylethanolamine derivatives, into liposomal membranes enables structural destabilization under low pH conditions, resulting in efficient drug release. Such systems have been investigated clinically for the targeted delivery of chemotherapeutics like doxorubicin [151] and vincristine [152], demonstrating improved tumor penetration and reduced off-target toxicity.

Similarly, redox-sensitive vesicles exploit the elevated intracellular concentrations of reducing agents, particularly glutathione, relative to extracellular fluids [88]. By integrating disulfide linkages within the lipid bilayer or polymer backbone, these vesicles undergo rapid structural breakdown upon entering reductive cytosolic environments, thereby releasing their payload directly into the cell interior. This mechanism is particularly valuable for enhancing the intracellular delivery of chemotherapeutics and nucleic acids, where precise cytosolic release is critical for efficacy [153,154].

Additional strategies include thermo- and photo-responsive systems, which rely on external stimuli to achieve controlled release. Thermosensitive vesicles, often incorporating lysolipids or specialized polymers, are engineered to destabilize and release their cargo when exposed to elevated temperatures, typically above 40–42 °C [36,43]. These systems can exploit localized hyperthermia at tumor sites or be activated by externally applied heating, offering synergistic potential in combination with hyperthermic therapies. On the other hand, photo-responsive vesicles incorporate light-sensitive chromophores that trigger structural disruption upon exposure to specific wavelengths [124]. Near-infrared (NIR) light, with its capacity to penetrate biological tissues, has been particularly effective for activating drug release at targeted sites, enabling precise spatial and temporal control while minimizing systemic exposure [155,156,157].

Together, these stimuli-responsive strategies highlight the increasing sophistication of vesicle-based delivery systems. By tailoring release to pathological microenvironments or applying external triggers, such designs advance the therapeutic window of vesicle-mediated treatments, enhance patient safety, and open new avenues for precision medicine and theranostic applications.

Enzyme-responsive vesicles are designed to degrade in the presence of specific enzymes, such as matrix metalloproteinases overexpressed in tumors or phospholipases in inflammatory sites. This strategy allows highly localized drug release and has been employed in preclinical cancer models [158].

### 8.4. Dual and Multi-Stimuli Vesicles, Clinical Applications, and Design Challenges

The emergence of dual- and multi-stimuli responsive vesicles represents one of the most innovative directions in the field of nanomedicine. Unlike conventional vesicles, which typically respond to a single environmental cue, these advanced systems are engineered to integrate multiple triggers, allowing them to achieve superior precision in drug delivery; some examples are summarized in Table 3. The rationale behind this approach lies in the complexity of pathological microenvironments, such as tumors or inflamed tissues, which often display a combination of biochemical and physical hallmarks. For example, tumors are simultaneously characterized by acidic extracellular pH, elevated intracellular glutathione levels, abnormal enzymatic activity, and in some cases local hyperthermia. By designing vesicles that respond to more than one of these conditions, drug release can be restricted to very specific sites and circumstances, thereby reducing off-target exposure and improving therapeutic outcomes [117,159,160].

A key advantage of dual- and multi-stimuli vesicles is their ability to integrate intrinsic biological cues with extrinsic, externally applied stimuli. pH- and redox-sensitive vesicles, for instance, exploit the acidic and reductive intracellular milieu of tumor cells, releasing their therapeutic cargo only once internalized, thus protecting healthy tissues during circulation. In contrast, thermo- and photo-responsive vesicles enable physicians to precisely dictate when and where drug release occurs by applying localized heating or near-infrared (NIR) light. Such systems not only provide spatiotemporal control but also allow combination with imaging technologies, moving toward theranostic applications that combine therapy and diagnostics within a single platform (Figure 1). This multidimensional control has positioned multi-stimuli vesicles as promising candidates for future precision medicine, where treatments can be tailored to the patient’s specific disease profile.

Concrete examples underscore the potential of these systems. The clinically approved PEGylated liposomal doxorubicin, Doxil^®^ (Caelyx^®^ in Europe) [41], illustrates how surface functionalization and passive targeting can significantly extend circulation half-life and enhance tumor accumulation. ThermoDox^®^ [161], a thermosensitive formulation of doxorubicin, demonstrates how vesicles can be combined with localized hyperthermia to achieve controlled release directly at tumor sites, and has progressed through multiple clinical trials, particularly for liver cancer. Preclinically, engineered extracellular vesicles functionalized with neuron-specific ligands have enabled delivery of siRNA across the blood–brain barrier, a longstanding challenge in neuropharmacology. Similarly, redox-sensitive polymersomes loaded with paclitaxel have shown superior cytotoxicity in tumor cells by ensuring that drug release occurs primarily in the intracellular compartment, thereby reducing systemic side effects. Together, these examples reveal the translational trajectory of stimuli-responsive vesicles, from basic design concepts to advanced clinical evaluation.

Nevertheless, despite encouraging progress, significant hurdles remain for their widespread clinical implementation. Functionalization and the incorporation of responsive materials add layers of complexity to manufacturing processes, raising both technical and regulatory concerns. One critical aspect is ligand density and orientation: excessive ligand coverage can interfere with vesicle stability or hinder receptor accessibility, while improper orientation reduces binding efficacy. Membrane stability must also be carefully balanced, as introducing responsive lipids or polymers may compromise structural integrity if formulations are not finely tuned. Immunogenicity presents another obstacle, particularly when protein- or antibody-based ligands are used, which may activate unwanted immune responses or accelerate clearance. Furthermore, the reproducible large-scale production of these systems under good manufacturing practice (GMP) standards remains challenging. Techniques such as click chemistry, maleimide conjugation, or lipid insertion are effective at the laboratory scale but require significant adaptation for industrial use without sacrificing reproducibility or cost-effectiveness. Future directions will likely involve integrating smart design strategies with advanced manufacturing technologies. Microfluidic platforms, for instance, offer promising solutions for achieving precise control over vesicle size, composition, and functionalization at scalable levels. At the same time, combining vesicle systems with advanced imaging tools could accelerate the development of theranostic formulations capable of real-time monitoring and tailored interventions. Importantly, regulatory frameworks will need to evolve to accommodate the unique complexity of multi-functional nanomedicines, ensuring both safety and efficacy.

In sum, dual- and multi-stimuli responsive vesicles epitomize the next generation of nanocarriers, uniting biology-inspired selectivity with engineered precision. Their continued development holds the potential not only to refine cancer therapy but also to expand into regenerative medicine, neurology, and infectious diseases, paving the way for personalized therapeutic strategies that are safer, more effective, and more adaptive to individual patient needs.

**Table 3:** Dual and Multi-Stimuli Vesicles, Clinical Applications, and Design Challenges [25,39,105,115,146,148,149,156,157,158,159,160,162,163,164,165,166].

Vesicle Type	Composition/Structural Features	Stimuli-Responsive Mechanisms (Single, Dual, Multi)	Representative Clinical/Biomedical Applications	Key Design Challenges
**Liposomes**	Phospholipid bilayers Tunable lamellarity Cholesterol for stability	**Single:** pH, temperature, light, redox (modified lipids) **Dual:** pH + temperature; pH + redox **Multi:** pH + light + enzymatic triggers (engineered formulations)	Drug delivery (oncology, antifungals, antibiotics) Vaccine platforms, Imaging agents	Leakage under stress Limited long-term stability Batch-to-batch reproducibility Rapid clearance without PEGylation
**Niosomes**	Non-ionic surfactants + cholesterol Stable membranes	**Single:** pH, temperature **Dual:** pH + redox **Multi:** pH + temperature + enzymatic degradation (polymer-modified)	Topical/transdermal delivery Gene delivery Antimicrobial formulations	Lower biocompatibility vs. liposomes Limited clinical penetration Scalability concerns
**Polymersomes**	Amphiphilic block copolymers Thick and robust membranes	**Single:** pH, temperature, redox, light **Dual:** pH + redox, temperature + redox **Multi:** pH + redox + magnetic/light triggers	Long-circulating drug carriers Gene and protein delivery Diagnostic imaging, hydrophobic drug encapsulation	Complex synthesis Potential polymer toxicity Slower regulatory acceptance Degradation kinetics must be tightly controlled
**Exosomes/Extracellular Vesicles**	Natural lipid–protein vesicles Composition determined by cell origin	**Single:** pH, redox gradients **Dual:** pH + enzymatic remodeling **Multi:** Engineering-dependent (surface ligands + pH + redox)	Regenerative medicine Inflammation modulation Intercellular communication Targeted delivery due to intrinsic tropism	Heterogeneity of isolates Low yield Purification challenges Limited standardization; Difficult large-scale production
**Hybrid Vesicles (Lipid–Polymer or Cell Membrane–Coated)**	Composite membranes combining lipid fluidity with polymer rigidity or biological membranes	**Single:** pH, redox, temperatura **Dual:** lipid (pH) + polymer (redox/thermal) **Multi:** pH + redox + light or thermal triggers	Long-circulating therapeutic carriers Targeted drug delivery (ligand-coated) Enhanced immune evasion	Fabrication complexity Reproducibility Ensuring stability of biological components Tuning dual/multi responsiveness consistently
**Micelles**	Amphiphilic surfactants or block copolymers; hydrophobic core	**Single:** pH, temperatura **Dual:** pH + redox, temperature + light **Multi:** pH + redox + enzymatic release	Solubilization of hydrophobic drugs Tumor-targeted delivery Controlled release systems	Rapid dissociation in vivo Limited cargo protection Require stabilizing strategies
**MOF- or Silica-Based Vesicle-Like Nanocarriers**	Hollow or porous inorganic/hybrid structures	**Single:** pH, redox, light, magnetic **Dual:** pH + redox, magnetic + thermal **Multi:** pH + redox + light or magnetic	Controlled release platforms Imaging contrast agents Combination therapy systems	Risk of metal ion toxicity Long-term biocompatibility unknown Degradation control in physiological conditions

## 9. Pharmacokinetics and Biodistribution of Vesicle-Based Drug Delivery Systems

Pharmacokinetics (PK) and biodistribution are fundamental determinants of the therapeutic efficacy and safety of vesicle-based drug delivery systems [111,167]. The physicochemical properties of vesicles—including size, surface charge, composition, and functionalization—strongly influence circulation half-life, tissue accumulation, cellular uptake, and clearance. Understanding these parameters is crucial for optimizing vesicle design, ensuring predictable delivery, and minimizing off-target effects.

The PK profile of vesicular carriers differs substantially from that of free drugs. Encapsulation within a vesicle can protect labile drugs from enzymatic degradation, reduce renal clearance, and modulate absorption. However, vesicles also interact with the mononuclear phagocyte system (MPS), serum proteins, and the endothelium, which collectively determine systemic distribution [168].

The pharmacokinetic behavior of vesicular nanocarriers is governed by a complex interplay of size, surface charge, composition, and interactions with biological components. These parameters collectively determine circulation half-life, tissue distribution, and clearance pathways, ultimately shaping therapeutic efficacy and safety.

Vesicle size is one of the most critical determinants of pharmacokinetics. Small unilamellar vesicles (20–100 nm) generally exhibit prolonged circulation, efficient tumor penetration via the enhanced permeability and retention (EPR) effect, and reduced uptake by the mononuclear phagocyte system (MPS). In contrast, larger vesicles > 200 nm are more prone to opsonization and clearance by Kupffer cells in the liver and macrophages in the spleen [169]. Polymersomes, with tunable membrane thickness and size, offer additional opportunities for modulating clearance rates and often achieve longer half-lives than conventional liposomes in preclinical studies. Techniques such as dynamic light scattering (DLS) and nanoparticle tracking analysis (NTA) are routinely employed to characterize vesicle size [170], while in vivo studies using radiolabeling or fluorescent dyes provide quantitative insights into biodistribution [171].

Surface charge, typically measured as zeta potential, also exerts a strong influence on vesicle fate [172]. Neutral or slightly negative vesicles tend to circulate longer and avoid rapid clearance by the MPS, while cationic vesicles, despite facilitating cellular uptake due to strong interactions with negatively charged membranes, are rapidly eliminated and may trigger immune activation. Zwitterionic coatings and PEGylation strategies mitigate these effects, conferring stealth properties while maintaining the capacity for active targeting via ligand conjugation. This charge-dependent behavior is particularly relevant in nucleic acid delivery, where cationic systems improve complexation with siRNA or mRNA but require careful optimization to balance efficacy and toxicity.

Another key determinant of vesicle behavior is the protein corona, formed upon exposure to plasma proteins. This dynamic layer, comprising both tightly bound “hard corona” proteins and loosely associated “soft corona” proteins, can either accelerate clearance or, in some cases, promote targeted delivery through receptor-mediated interactions [173]. Strategies such as PEGylation [42,86,152], zwitterionic polymers [133], or the use of extracellular vesicle (EV) membranes help to minimize unfavorable protein adsorption, thereby enhancing circulation time and improving pharmacokinetic profiles [1,98,139,173].

Tissue distribution is shaped by both passive and active targeting mechanisms. Through the EPR effect, vesicles accumulate preferentially in tumors and inflamed tissues, whereas active targeting strategies employ ligands or antibodies to enhance cellular specificity. For example, liposomes functionalized with anti-HER2 antibodies concentrate in HER2-positive breast tumors [174], RGD-conjugated vesicles target integrins in tumor vasculature [134], and engineered exosomes bearing neuron-specific ligands successfully traverse the blood–brain barrier to deliver siRNA to neuronal tissue [120]. Critical parameters such as vesicle size, ligand density, and surface chemistry must be finely tuned to maximize tissue penetration while minimizing off-target accumulation.

The metabolic fate and clearance pathways of vesicular nanocarriers largely depend on their composition and physicochemical properties [131]. Lipid-based systems, such as liposomes and niosomes, are primarily degraded by phospholipases and subsequently integrated into the body’s endogenous lipid metabolism [175,176]. In contrast, polymer-based vesicles undergo hydrolysis, enzymatic degradation, or redox-responsive disassembly, depending on the chemical nature of their constituent polymers [177]. Extracellular vesicles (EVs) are generally internalized through endocytosis and subsequently processed within lysosomal compartments [178]. Due to size-dependent filtration in the kidneys, renal clearance is typically limited to particles smaller than 5–10 nm, whereas hepatic and biliary excretion constitute the predominant elimination routes for most therapeutic vesicle formulations.

Advanced imaging and tracking techniques have become essential tools for understanding vesicle pharmacokinetics in vivo. Fluorescent dyes such as DiR and DiD enable optical imaging, while radiolabeled isotopes (e.g., ^99^ᵐTc, ^111^In) facilitate quantitative biodistribution studies using PET or SPECT. Magnetic resonance imaging (MRI) contrast agents encapsulated within vesicles provide anatomical and functional insights, supporting both preclinical and clinical evaluations. These approaches allow real-time monitoring of circulation, tissue accumulation, and clearance, thereby guiding rational design and optimization of vesicular carriers [179].

Clinically, pharmacokinetic modulation has been crucial to the success of several formulations. Doxil^®^/Caelyx^®^ reduces cardiotoxicity by prolonging circulation and enhancing tumor uptake [15,41], while AmBisome^®^, a liposomal amphotericin B formulation, minimizes nephrotoxicity by altering biodistribution relative to free drug [78]. Emerging EV-based therapies, now in early clinical trials, highlight the promise of naturally derived vesicles for targeted delivery of siRNA and chemotherapeutics, achieving tissue-specific effects with minimal immune activation [90,135].

Despite these advances, translating vesicular nanocarriers into clinical applications remains a major challenge. Interpatient variability in the enhanced permeability and retention (EPR) effect, along with differences in protein corona composition and mononuclear phagocyte system (MPS) activity, often results in inconsistent therapeutic outcomes. Consequently, the integration of individualized pharmacokinetic profiling and predictive computational modeling has gained increasing attention as a strategy to bridge preclinical and clinical performance. Ultimately, the goal is to align vesicle design with patient-specific biological parameters, thereby optimizing pharmacokinetics, biodistribution, and overall therapeutic efficacy while ensuring maximal safety and reproducibility in clinical settings [180,181].

## 10. Applications in Biomedicine and Personalized Care

Vesicle-based drug delivery systems have transformed biomedical therapeutics by providing highly versatile platforms capable of encapsulating small molecules, biologics, nucleic acids, and imaging agents. Their design allows for precise control over pharmacokinetics, biodistribution, and intracellular delivery, enabling applications across oncology, neurology, infectious diseases, regenerative medicine, and vaccines. By combining passive targeting through the Enhanced Permeability and Retention (EPR) effect with active targeting via ligand or antibody functionalization, vesicles offer controlled release and site-specific delivery that traditional formulations cannot achieve [34,115,118,182,183].

In oncology, vesicles such as liposomes, polymersomes, and extracellular vesicles (EVs) have been extensively explored to improve chemotherapeutic efficacy while minimizing systemic toxicity [12,19,25,39,43,46,57,80,81,86,88,89,91,94,95,105,109,116,134,139,142,143,152,160,174,180]. PEGylated liposomal doxorubicin, for example, reduces cardiotoxicity and achieves prolonged tumor accumulation, while liposomal paclitaxel improves solubility and avoids hypersensitivity reactions, enabling higher dosing [184]. Stimuli-responsive polymersomes exploit tumor-specific intracellular conditions, such as elevated glutathione levels, to trigger intracellular drug release [114]. Vesicles also facilitate the delivery of nucleic acids, protecting siRNA [90,135], mRNA [166], or CRISPR/Cas components from enzymatic degradation and enhancing cellular uptake [163], even across challenging barriers such as the blood–brain barrier. Functionalization with tumor-targeting ligands [159] or antibodies further enhances selective accumulation, reducing off-target toxicity and improving therapeutic outcomes, as demonstrated in numerous preclinical models showing tumor regression and improved survival [80].

Neurological applications highlight the unique ability of vesicles to overcome delivery barriers in the central nervous system [117]. Exosomes and polymersomes functionalized with ligands such as transferrin or lactoferrin can penetrate the blood–brain barrier, delivering neurotherapeutics to neurons and glial cells [185]. Preclinical studies show that neuron-targeted EVs can deliver siRNA or neurotrophic factors, offering potential interventions for neurodegenerative diseases, brain tumors, and stroke [33,112]. Stimuli-responsive vesicles provide controlled intracellular release, enabling precise temporal and spatial regulation of therapeutic agents within neural tissue.

Vesicle-based systems have also demonstrated significant benefits in infectious disease treatment. Liposomal formulations, such as AmBisome^®^, improve the safety profile of amphotericin B while maintaining efficacy against systemic fungal infections [78,186]. Encapsulation of antibiotics like vancomycin or daptomycin enhances pharmacokinetics and intracellular delivery, increasing efficacy against intracellular pathogens [187,188]. EVs carrying bacterial or viral antigens have shown promise as vaccine platforms, stimulating robust immune responses through natural immunogenicity and biocompatibility [189]. Liposomes and niosomes have been explored for antiviral drug delivery, enhancing bioavailability and reducing off-target effects [190], while EVs can deliver antiviral siRNA directly to infected cells with minimal immune activation [191].

In regenerative medicine, vesicle-based platforms facilitate the delivery of growth factors, cytokines, and nucleic acids to promote tissue repair and regeneration. MSC-derived EVs transport microRNAs and proteins that support cardiac repair post-myocardial infarction and enhance angiogenesis [192]. Hydrogel-integrated liposomes allow controlled release of growth factors for bone and cartilage repair, while polymersomes provide sustained delivery of osteogenic and neurogenic factors to targeted tissues [130]. These strategies combine biocompatibility with precise spatial and temporal control, improving the efficiency of regenerative therapies.

Vesicles also enable combination therapies, allowing the co-delivery of multiple therapeutic agents such as chemotherapeutics with gene therapies or immunomodulators. Dual-drug liposomes encapsulating doxorubicin and cisplatin enhance cytotoxic synergy in tumors [193], polymersomes can co-deliver immune checkpoint inhibitors alongside chemotherapeutics for enhanced antitumor immunity [119], and EVs can simultaneously modulate the tumor microenvironment and deliver regenerative or genetic payloads [95].

Clinical translation of vesicle-based therapies is already evident, with approvals such as Doxil^®^ [15,41], AmBisome^®^ [78,186], and liposomal irinotecan (Onivyde^®^) [79] demonstrating their application in routine medical practice. Preclinical studies continue to expand the potential of these systems, including EV-mediated siRNA delivery across the blood–brain barrier, polymersome-controlled chemotherapeutic release, and niosome-based vaccines. However, challenges remain, including patient variability in EPR-mediated tumor targeting, biodistribution heterogeneity, immunogenicity, and the need for scalable manufacturing processes that maintain vesicle integrity and bioactivity. Regulatory compliance, sterility, reproducibility, and quality control are essential for successful clinical translation, as is balancing targeted delivery with minimal systemic toxicity [194].

Overall, vesicle-based drug delivery systems (Table 1) provide a versatile and highly adaptable platform for precision medicine. Their ability to enhance therapeutic efficacy, reduce adverse effects, and deliver complex biologics positions them as critical tools in oncology, neurology, infectious diseases, regenerative medicine, and vaccinology. Ongoing developments in material design, functionalization strategies, and controlled release mechanisms promise to expand the impact of vesicle-based therapies, bridging preclinical research and clinical application for a wide range of biomedical challenges; some results were summarized in Table 4.

## 11. Theranostic Vesicles: Integrating Therapy and Diagnosis

**Figure 3:**
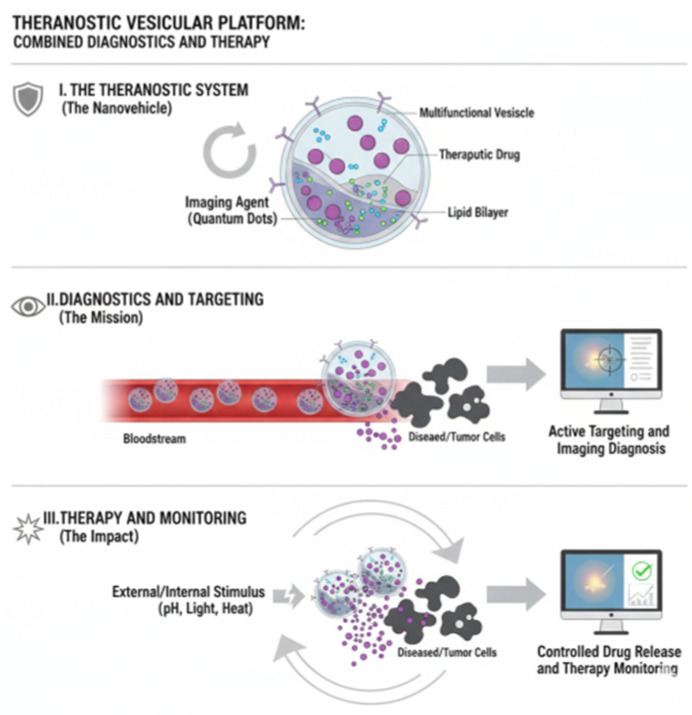
Theranostics is a personalized medicine strategy that integrates a diagnostic tool with a therapeutic agent into a single approach, enabling the visualization of a target before its treatment. The process involves first using a traceable agent (e.g., a radiotracer) to confirm the presence and localization of the disease target via imaging (Diagnosis), and then administering a coupled powerful agent (e.g., a therapeutic radioisotope) to selectively destroy the identified tissue (Therapy). This approach drastically enhances treatment efficacy and patient selection while simultaneously minimizing systemic toxicity to healthy tissues, defining a true precision medicine paradigm.

The concept of theranostics, which integrates therapeutic and diagnostic functions within a single nanoscale platform, has gained remarkable attention in recent years, and vesicular systems are at the forefront of this innovation (Figure 3) [12,37,108,119,144,190,195,196,197]. Their ability to encapsulate diverse cargoes, modulate surface chemistry, and maintain biocompatibility makes them ideally suited for the dual purpose of delivering treatment while simultaneously enabling real-time monitoring of drug distribution and therapeutic response. By combining imaging agents with therapeutic payloads, theranostic vesicles allow clinicians and researchers to visualize where and how drugs are delivered, track their biodistribution, and assess efficacy non-invasively, thereby advancing the paradigm of personalized medicine.

A wide range of imaging modalities has been incorporated into vesicular platforms to support these applications. Liposomes or polymersomes labeled with near-infrared fluorescent dyes permit in vivo tracking and longitudinal monitoring of biodistribution, while vesicles encapsulating gadolinium or superparamagnetic iron oxide nanoparticles (SPIONs) serve as contrast-enhanced probes for magnetic resonance imaging (MRI), providing both anatomical and functional insights [198]. Similarly, vesicles radiolabeled with isotopes such as ^99^ᵐTc [199] or ^111^In [200] enable highly sensitive positron emission tomography (PET) or single-photon emission computed tomography (SPECT), offering quantitative readouts of accumulation and pharmacokinetics in real time. These imaging strategies are particularly valuable in oncology, where accurate detection of tumor uptake and early signs of therapeutic response are critical for clinical decision-making.

Beyond imaging, theranostic vesicles co-deliver therapeutic agents alongside diagnostic markers. For instance, liposomal doxorubicin formulations have been combined with fluorescent tracers or radioisotopes to enable simultaneous chemotherapy and imaging-guided monitoring of tumor progression [201]. In photothermal therapy, liposomes or polymersomes carrying gold nanorods [80] or infrared dyes not only provide imaging contrast but also generate localized hyperthermia upon near-infrared irradiation, resulting in precise ablation of tumor tissues [165]. Gene therapy applications have also benefited from this approach: extracellular vesicles and cationic liposomes functionalized with small interfering RNAs (siRNAs) and labeled with fluorescent probes have been used to track intracellular delivery while achieving gene silencing, offering a powerful tool for treating genetic and oncological diseases [202].

Both passive and active targeting strategies contribute significantly to the success of vesicle-based theranostics [203,204,205]. Passive targeting leverages the enhanced permeability and retention (EPR) effect to accumulate vesicles in tumors with leaky vasculature, while active targeting introduces specificity by decorating vesicles with ligands, antibodies, peptides, or aptamers that bind to receptors overexpressed on diseased cells. The integration of targeting strategies with imaging enables a dynamic understanding of therapeutic distribution, allowing clinicians to optimize dosing, detect off-target accumulation at an early stage, and evaluate treatment efficacy with greater precision.

Preclinical and early clinical investigations increasingly demonstrate the potential of vesicular theranostic systems to integrate diagnosis and treatment within a single platform. Doxil-derived formulations have been adapted to visualize tumor accumulation and therapeutic efficacy in real time [129,164], while SPION-loaded liposomes enable MRI-guided chemotherapy, allowing precise tumor delineation and controlled drug release [206,207]. Likewise, engineered exosomes carrying siRNA and near-infrared dyes have successfully silenced oncogenic targets and monitored biodistribution in animal models [121,208]. Polymersomes incorporating gold nanorods further exemplify the versatility of these systems by combining imaging functions with photothermal tumor ablation, underscoring their promise in precision oncology [209,210].

Theranostic vesicles offer remarkable advantages, providing non-invasive, real-time tracking of drug delivery, enabling dose optimization, and advancing personalized therapy by directly linking diagnostic information with therapeutic response. However, their clinical translation still faces significant challenges. The simultaneous encapsulation of imaging and therapeutic agents increases formulation complexity and may compromise stability, while their dual functionality imposes stringent regulatory demands, requiring comprehensive validation of both diagnostic and therapeutic performance. In addition, potential toxicities from imaging components—particularly those involving heavy metals or photothermal nanomaterials—necessitate careful evaluation and mitigation strategies. Looking ahead, research is expanding the functional and analytical potential of these systems. The development of multimodal vesicles integrating MRI, PET, and fluorescence imaging could support more comprehensive diagnosis and therapy monitoring. Stimuli-responsive carriers activated by light, heat, or pH changes promise precise spatiotemporal control over drug release. Moreover, the integration of theranostic data with artificial intelligence and advanced image analysis may enable real-time prediction of therapeutic outcomes and adaptive dosing. Hybrid systems derived from extracellular vesicles, combining natural targeting capabilities with excellent biocompatibility, are also emerging as next-generation platforms that unite synthetic and biological nanocarriers.

By merging therapeutic delivery with diagnostic insight, vesicular theranostics are transforming the field of nanomedicine, creating systems capable not only of treating disease but also of guiding, monitoring, and adapting therapy, bringing truly personalized medicine closer to clinical realization [128].

## 12. Regulatory, Safety, and Manufacturing

The successful translation of vesicle-based drug delivery systems from the research laboratory to clinical practice requires careful navigation of multiple, interdependent factors, including adherence to stringent regulatory frameworks, comprehensive demonstration of safety and biocompatibility, and the development of scalable, reproducible manufacturing processes. Regulatory authorities such as the U.S. Food and Drug Administration (FDA) and the European Medicines Agency (EMA) play a pivotal role in this translational pathway, situating vesicle-based therapeutics within broader categories of biological products and nanomedicines [10,21,194,211]. Their evaluation emphasizes not only quality, safety, and efficacy but also the reproducibility and robustness of production methods, which are considered essential for long-term clinical viability.

The regulatory classification of vesicular systems, an essential early step in this process, is summarized in Table 5. Depending on their composition and intended use, they may be defined as drug products, such as liposomes, niosomes, and polymersomes encapsulating small molecules or biologics; as biologics, in the case of extracellular vesicle (EV)-based therapeutics or genetically engineered vesicles; or as combination products, exemplified by theranostic vesicles that integrate diagnostic and therapeutic functionalities. This categorization is not merely administrative; it dictates the applicable regulatory pathway, the scope of preclinical studies required, and the ultimate approval process. Consequently, researchers and developers are compelled to consider classification issues from the earliest stages of design to avoid delays or complications in later phases of translation.

International guidelines and standards have been established to support this complex landscape. The principles of Quality by Design (QbD) [214,215], articulated in ICH guidelines Q8–Q11 [216], emphasize a systematic development approach that links product performance to critical quality attributes (CQAs), such as vesicle size distribution, encapsulation efficiency, stability of the therapeutic payload, and the presence of specific surface functionalities. Meanwhile, standards issued by ISO and USP provide further guidance on characterization requirements, sterility assurance, and endotoxin control, which are indispensable for ensuring patient safety [217]. The EMA has also published reflection papers specifically addressing nanomedicines, including liposomal and polymeric formulations, providing additional frameworks for developers [212,213]. Adherence to these standards is indispensable, not only to achieve regulatory approval but also to ensure reproducibility across different manufacturing sites and long-term clinical scalability.

Safety and toxicology remain central considerations in the translation of vesicular systems [218]. Immunogenicity is a well-documented concern, particularly for cationic liposomes or EVs carrying proteins derived from non-human sources. Approaches such as PEGylation and the use of stealth coatings are routinely employed to prolong circulation times by evading recognition and clearance by the mononuclear phagocyte system. However, these modifications are not without drawbacks, as the emergence of anti-PEG antibodies in some patients has been reported, potentially reducing therapeutic efficacy or causing hypersensitivity reactions [55,219,220]. Beyond immunogenicity, cytotoxicity must be rigorously assessed, as it can be influenced by the physicochemical composition of the vesicle, its surface charge, and the properties of the encapsulated cargo. Biocompatibility of constituent lipids, surfactants, or polymers is essential, and degradation products must be carefully evaluated to ensure that they neither accumulate in tissues nor elicit toxic responses. Preclinical toxicological studies, therefore, extend beyond general cytotoxicity to include organ-specific evaluations, hematological and cytokine profiling, and assessments of inflammatory pathways [221]. Another dimension of safety involves unintended off-target effects, which may arise when targeting ligands exhibit cross-reactivity with non-diseased tissues [222,223]. Advanced strategies, such as stimuli-responsive vesicular membranes, finely tuned ligand engineering, and controlled release technologies, are being developed to mitigate these risks and enhance therapeutic precision.

The manufacturing of vesicle-based therapeutics represents another major pillar in their clinical translation. Compliance with Good Manufacturing Practices (GMP) is essential to ensure sterility, consistency, and reproducibility [224]. This involves the establishment of controlled cleanroom environments, validated aseptic processing methods, sterile filtration procedures, and robust quality control systems capable of identifying batch-to-batch variations. Recent advances in scalable production technologies have further enabled clinical application. For example, liposomes and niosomes can be produced using microfluidics, extrusion, or ethanol injection techniques, each of which has been adapted for GMP compliance [113]. Polymersomes, by contrast, are typically manufactured via solvent displacement or emulsion-based methods, while extracellular vesicles require more complex isolation techniques, such as tangential flow filtration, ultracentrifugation, and chromatographic approaches [225]. To guarantee quality, a broad set of analytical methods is employed to monitor critical parameters, including vesicle size, polydispersity, zeta potential, encapsulation efficiency, sterility, endotoxin content, and stability. Techniques such as dynamic light scattering (DLS), transmission electron microscopy (TEM), high-performance liquid chromatography (HPLC), and immunoassays are routinely integrated into release criteria and long-term stability testing [226,227].

Despite these advances, significant challenges remain. The inherent complexity of multifunctional vesicular systems, particularly those designed for theranostic applications or functionalized with targeting ligands, requires unprecedented levels of characterization and standardization. Patient variability represents another barrier, as interindividual differences in mononuclear phagocyte system activity, protein corona formation, and the magnitude of the enhanced permeability and retention (EPR) effect can dramatically influence therapeutic outcomes. Regulatory uncertainty further complicates the landscape, especially for emerging EV-based therapeutics, where definitions, potency assays, and regulatory pathways are still evolving. Moreover, the cost and logistical demands associated with large-scale manufacturing of complex vesicular systems impose additional hurdles, limiting their widespread adoption.

Looking toward the future, several promising directions are emerging. Automated, closed-system manufacturing platforms based on microfluidics or continuous-flow processing are expected to reduce variability, improve scalability, and minimize contamination risks. The application of advanced analytical tools, including high-resolution imaging, nanoparticle tracking analysis, and omics-based profiling, is enhancing the precision of vesicle characterization and the understanding of their biological behavior. In the case of extracellular vesicles, the development and global harmonization of standardized potency assays will be crucial to ensuring reliable and reproducible therapeutic effects. Finally, the integration of vesicular systems with personalized medicine paradigms offers perhaps the most transformative potential. Predictive pharmacokinetic and pharmacodynamic models, combined with theranostic vesicles tailored to individual patient physiology, open the possibility of truly customized therapeutic interventions, bridging the gap between nanomedicine innovation and clinical utility.

In sum, the clinical translation of vesicle-based drug delivery systems is a highly multidisciplinary endeavor that requires the convergence of regulatory science, toxicology, pharmaceutical engineering, and systems biology. While significant obstacles remain, the ongoing evolution of regulatory frameworks, the refinement of scalable manufacturing technologies, and the integration of advanced characterization methods collectively pave the way for the emergence of vesicle-based therapeutics as a cornerstone of next-generation precision medicine.

## 13. Translational Landscape and Technology Readiness of Smart Vesicles

To contextualize the current maturity of the different smart vesicle platforms, we include here a comparative overview of their technology readiness levels (TRLs), approved clinical products, and representative ongoing clinical trials (Table 6). Overall, natural vesicles such as extracellular vesicles (EVs) and liposomes currently exhibit the highest translational maturity, with several liposomal formulations already approved for clinical use. In contrast, biomimetic and fully synthetic vesicles—while technologically promising—remain mostly at early-to-intermediate TRLs due to pending optimization of large-scale production, stability, and regulatory standardization.

## 14. Future Perspectives and Concluding Remarks

Vesicle-based drug delivery systems have revolutionized therapeutic strategies across oncology, neurology, infectious diseases, regenerative medicine, and vaccination. The incorporation of advanced functionalization techniques, stimuli-responsive release mechanisms, and theranostic integration has provided unprecedented control over drug biodistribution, cellular targeting, and real-time monitoring of therapeutic response. While clinical progress has been remarkable, significant challenges remain in achieving consistent translation, navigating regulatory frameworks, and scaling up manufacturing processes. Looking ahead, several emerging trends and technological innovations are shaping the future landscape of vesicle-based therapeutics, while ongoing challenges continue to define the path toward clinical adoption. Artificial intelligence and machine learning are poised to play a transformative role in this field. By leveraging predictive modeling, AI can optimize vesicle formulation, identify lipid or polymer compositions that enhance stability, and guide the density and orientation of ligands for maximal receptor binding. Such tools can also predict biodistribution patterns tailored to patient-specific physiology. When combined with real-time imaging from theranostic vesicles, these computational advances open the door to adaptive dosing strategies and highly personalized treatment plans. At the material level, hybrid vesicles represent an important direction for innovation. By combining lipids, polymers, and extracellular vesicle components, these hybrid systems aim to merge the circulation stability and tunability of synthetic platforms with the natural targeting and biocompatibility of biological membranes. Similarly, multifunctional and modular vesicles are being developed to carry diverse combinations of therapeutic drugs, nucleic acids, imaging agents, and responsive elements. Such modularity enables customization for specific diseases, ensuring that targeting, controlled release, and theranostic monitoring can be integrated into a single, adaptable platform.

Extracellular vesicle (EV) engineering is another area gaining momentum. Due to their intrinsic compatibility with human physiology, natural targeting properties, and ability to cross biological barriers such as the blood–brain barrier, EVs are particularly promising for applications in oncology, central nervous system disorders, and regenerative medicine. Advances in genetic manipulation, surface modification, and scalable isolation technologies are expected to accelerate their transition from preclinical research into clinical use.

These developments are closely aligned with the principles of personalized medicine. Vesicle-based therapeutics allow for patient-specific targeting and dosing strategies, while theranostic-enabled imaging provides clinicians with immediate feedback on biodistribution, therapeutic efficacy, and potential off-target effects. When integrated with genomic, proteomic, and metabolomic data, vesicles offer the possibility of tailoring therapies not only to disease states but also to individual biological profiles, moving closer to truly personalized healthcare. Despite these opportunities, several challenges remain unresolved. The heterogeneity of the enhanced permeability and retention (EPR) effect limits the reliability of passive targeting, while protein corona formation can alter vesicle circulation time and targeting specificity. Immune responses, particularly against PEGylated or exogenously functionalized vesicles, present additional hurdles. Manufacturing remains a bottleneck, as GMP-compliant production of complex, multifunctional, or hybrid vesicles requires high resource investment and technical sophistication. Promising solutions are being developed, including AI-guided design, novel functionalization strategies, modular and scalable manufacturing processes, and advanced preclinical models that better predict human pharmacokinetics and biodistribution. The regulatory landscape is also evolving in response to these emerging technologies. Regulatory agencies are beginning to address the complexities of multifunctional and theranostic vesicles, particularly extracellular vesicle–derived systems. Harmonization of standards for potency, characterization, and quality control will be essential to facilitate translation. Early and continuous dialogue with regulators, combined with rigorous preclinical validation, will be crucial to ensure safe and efficient clinical approval.

In summary, vesicle-based drug delivery systems embody a powerful and versatile platform for modern therapeutics. Liposomes, niosomes, polymersomes, and extracellular vesicles each provide unique advantages in encapsulation, targeting, and controlled release. Functionalization strategies, stimuli-responsive mechanisms, and theranostic integration add further layers of precision, enabling therapies that are both effective and patient-specific while minimizing systemic toxicity. Continued progress in hybrid vesicles, EV engineering, AI-guided optimization, and multimodal theranostics will expand the range of clinical applications and bring vesicle-based systems closer to becoming mainstream therapies. Overcoming challenges in reproducibility, immunogenicity, scalability, and regulation will be the key to achieving broader adoption. Ultimately, these systems are poised to remain a cornerstone of personalized medicine, bridging nanotechnology and clinical practice to provide safer, more effective and adaptable treatments for the future of healthcare.

## Figures and Tables

**Table 1:** Comparative analysis of main vesicle types [2,8,13,14,18,19,21,25,26,27,28,29,30,31,32,33,34,35,36,37].

Vesicle Type	Structural Features	Functional Attributes	Advantages	Limitations	Performance in Delivery
**Liposomes**	Phospholipid bilayers Tunable lamellarity Encapsulate hydrophilic (core) and hydrophobic (membrane) drugs.	High biocompatibility Modifiable surface Responsive to pH or T	FDA-approved precedents Scalable production Well-defined composition	Prone to leakage Limited mechanical stability Rapid clearance without PEGylation	Good carriers for antibiotics Effective for localized delivery but moderate retention in bone microenvironment
**Polymeric vesicles (polymersomes)**	Amphiphilic block copolymers forming robust bilayers Thicker membranes than liposomes	High structural stability Controlled permeability Slow Sustained release	Superior mechanical strength Customizable degradation Suitable for high-load cargo	More complex synthesis Potential cytotoxicity depending on polymer	Ideal for sustained release in chronic infections Enhanced durability under inflammatory conditions
**Extracellular vesicles (exosomes)**	Natural lipid–protein vesicles (30–150 nm) Enriched in signaling molecules	Intrinsic targeting Minimal immunogenicity Participate in regeneration pathways	Excellent biocompatibility Innate cell communication; Improved bone regeneration	Heterogeneity of preparations Low yield challenging large-scale production	Strong potential for delivering anti-inflammatory cues Best regenerative profile but limited standardization
**Hybrid vesicles (lipid–polymer or cell membrane -coated)**	Composite structures combining lipids with polymers or natural membranes	Synergistic properties: stability + biointerfacing Tunable targeting	Improved circulation Reduced immune clearance Enhanced control over drug kinetics	Complex fabrication Reproducibility concerns	Highly promising for theranostic systems Improved targeting to bone infection sites
**Metal—organic framework (MOF) nano-vesicles**	Porous crystalline scaffolds capable of forming hollow or vesicle-like structures	Exceptional loading capacity pH-triggered release	High stability Strong protection of cargo; Large metal nanoparticle encapsulation capability	Potential metal ion toxicity Limited long-term biocompatibility data	Effective antimicrobial performance Excellent for controlled release but still preclinical

**Table 2:** Comparative Overview of Vesicular Drug Delivery Systems [3,12,16,20,21,22,24,25,27,29,38,39,43,44,45,46,53,56,81,85,88,95,101,106,107,108,111,112,113,114,115,116,117,118,119,120,121,122,123,124,125,126,127,128,129,130,131].

Vesicle Type	Composition	Key Properties	Preparation Methods	Applications	Advantages	Challenges
**Liposomes**	Phospholipid bilayers ± Cholesterol	Encapsulate hydrophilic drugs in aqueous core Hydrophobic drugs in membrane	Thin-film hydration Ethanol injection Reverse-phase evaporation Microfluidics Remote loading	Oncology (Doxil) Antifungal therapy (AmBisome) Pain management	Clinically validated Biocompatible Versatile formulations	Limited stability Oxidative degradation Rapid clearance without PEGylation
**Niosomes**	Non-ionic surfactants (Span, Tween) ± Cholesterol	High chemical/physical stability Lower cost Reduced oxidative susceptibility	Thin-film hydration Reverse-phase evaporation Microfluidics	Dermal delivery Ocular, oral, and parenteral delivery Vaccines	Cost-effective Stable Adaptable to transdermal systems	Less biocompatible than lipids Regulatory acceptance still limited
**Polymersomes**	Amphiphilic block copolymers	Thicker, robust membranes Tunable permeability Stimuli -responsive	Self-assembly Microfluidic mixing Solvent exchange	Gene delivery Anticancer therapy Immunotherapy	Mechanical strength Extended circulation Programmable release	Clinical translation limited Scalability and polymer toxicity concerns
**Extracellular Vesicles (EVs)/Exosomes**	Naturally secreted nanoscale vesicles (lipids, proteins, nucleic acids)	Intrinsic targeting ligands Low immunogenicity Cross biological barriers	Ultracentrifugation Density gradients SEC Tangential flow Immunocapture	Oncology Regenerative medicine Vaccine delivery	Natural targeting Low immune activation Blood–brain barrier crossing	Manufacturing scalability Reproducibility Potency assays
**Hybrid and Specialized Vesicles**	Combinations of natural and synthetic systems (liposome–EV, polymersome–lipid, niosome–lipid) Functionalized constructs (magnetoliposomes, immunoliposomes)	Tailored balance of stability, targeting, and functionality Can integrate stimuli-responsiveness	Hybridization of lipids, polymers, surfactants, and/or inorganic nanoparticles	Oncology Theranostics Cardiovascular disease Regenerative medicine	Combine best features of multiple systems Multifunctionality Theranostic potential	Complex synthesis Scale-up challenges Regulatory hurdles

**Table 4:** Biomedical Applications of Vesicle-Based Drug Delivery Systems: Vesicle Types, Functions, Examples, Advantages, and Challenges [3,9,16,21,48,84,107,190].

Biomedical Area	Vesicle Types	Objectives/Function	Examples/Applications	Advantages	Challenges
**Oncology**	Liposomes, Polymersomes, EVs	Delivery of chemotherapeutics and nucleic acids Targeted therapies via ligands or antibodies	Doxil^®^: PEGylated liposomal doxorubicin Liposomal paclitaxel Redox-sensitive polymersomes EVs carrying siRNA or CRISPR components	Reduced systemic toxicity Enhanced tumor accumulation Potential for active targeting	Patient variability in EPR effect Tumor heterogeneity Scalable manufacturing
**Neurology/CNS**	EVs, Polymersomes, PEGylated liposomes	Cross the blood–brain barrier Deliver neurotherapeutics	Neuron-targeted exosomes delivering siRNA PEGylated liposomes with transferrin/lactoferrin ligands Stimuli-responsive polymersomes	CNS penetration Specific intracellular delivery Potential for neurodegenerative diseases and brain tumors	Restrictive blood–brain barrier Possible immunogenicity Precise control of release
**Infectious Diseases**	Liposomes, EVs, Niosomes	Improve stability and bioavailability of antimicrobials/antivirals Vaccine delivery	AmBisome^®^: liposomal amphotericin B Liposomal vancomycin/daptomycin EV-based vaccines carrying bacterial or viral antigens	Reduced toxicity Targeted delivery Enhanced immune responses	Standardization of vesicle-based vaccines Variability in immune response Scalable production
**Regenerative Medicine**	EVs, Liposomes, Polymersomes	Delivery of growth factors, cytokines, and microRNAs Promote tissue repair	MSC-derived EVs for cardiac repair and angiogenesis Hydrogel-integrated liposomes for bone and cartilage Polymersomes for osteogenic and neurogenic factors	Sustained and localized release Biocompatibility Enhanced tissue regeneration	Precise dose and release control Stability of biomolecule loaded vesicles Scalability and regulatory compliance
**Vaccines**	Liposomes, Niosomes, EVs	Antigen protection; Enhance immunogenicity Flexible administration	Liposomal vaccines: hepatitis A, influenza Niosome-based vaccines: mucosal delivery EV-based vaccines: tumor or viral antigens	Antigen protection Potentiated immune response Potential for single-dose immunization	Storage stability Variability in immune response Large-scale production and regulation
**Combination Therapies**	Liposomes, Polymersomes, EVs	Co-delivery of multiple agents Therapeutic synergy Microenvironment modulation	Dual-drug liposomes: doxorubicin/+ cisplatin Polymersomes: chemotherapy + immunotherapy EVs: nucleic acids + small-molecule drugs	Synergistic therapeutic effects Reduced drug resistance Design flexibility	Formulation complexity Controlled release of multiple agents Scalability and regulatory challenges

**Table 5:** Summary of Regulatory, Safety, and Manufacturing Considerations for Vesicle-Based Therapeutics [10,21,194,212,213].

Vesicle Type	Regulatory Considerations	Safety Concerns	Manufacturing Challenges
**Liposomes**	Several FDA/EMA-approved products (e.g., Doxil, AmBisome) Clear regulatory pathways exist.	Generally safe PEGylation may cause immune reactions (“accelerated blood clearance”) Cardiotoxicity reduction compared to free drugs.	Scalable GMP processes established, but maintaining uniform size, lamellarity, and stability is critical.
**Polymersomes**	Limited clinical translation Need for standardized characterization and toxicity profiling Regulatory framework less defined.	Polymer degradation products may induce toxicity Long-term safety not fully established.	Reproducibility in polymer synthesis, batch consistency, and scale-up remain challenges.
**Niosomes**	Few clinical studies Regulatory pathways less mature than liposomes Require more safety validation.	Surfactant toxicity and stability issues can affect tolerability.	Large-scale production less developed Stability optimization needed.
**Extracellular Vesicles (EVs)**	Highly complex regulatory landscape Classification varies (biologic vs. drug vs. advanced therapy medicinal product).	Risk of immune activation or transmission of unwanted biomolecules Heterogeneity complicates safety evaluation.	Standardized isolation, purification, and scalable GMP production are major bottlenecks.
**Hybrid Vesicles (lipid–polymer, liposome–EV)**	No established regulatory framework Considered highly complex products needing case-by-case evaluation.	Possible immunogenicity from mixed components Unclear long-term safety.	Manufacturing reproducibility is difficult Hybrid composition complicates QC and regulatory approval.
**Stimuli-Responsive Vesicles**	Regulatory agencies require extensive stability and safety testing under variable conditions (pH, redox, temperature).	Risk of premature release or off-target activation Unknown effects of responsive materials in humans.	Scalability is difficult Maintaining responsiveness while ensuring stability during storage and transport is challenging.
**Theranostic Vesicles**	Dual-function nature complicates approval Must meet requirements for both drugs and diagnostics.	Toxicity from imaging agents (e.g., gadolinium, SPIONs) Balance needed between diagnostic and therapeutic payloads.	Co-encapsulation and stability of multiple cargos are technically complex Regulatory submissions are more demanding.

**Table 6:** Translational Status of Vesicle-Based Delivery Systems [7,228,229,230,231,232,233].

Vesicle Type	Approximate TRL Range	Approved Products (Examples)	Representative Clinical Trials (NCT/Estudio)	Remarks/Considerations
**Liposomes (conventional/PEGylated/stimuli-sensitive)**	TRL 8–9 (clinically established)	Doxil/Caelyx (liposomal doxorubicin) anticancer AmBisome (liposomal amphotericin B) antifúngico Onivyde (liposomal irinotecan) Liposomal vaccines/mRNA lipids (e.g., for COVID-19)	NCT03088813. Irinotecan liposome (Onivyde) versus Topotecan in small-cell lung cancer (Phase III) NCT02562378. Trastuzumab + non-PEGylated liposomal doxorubicin in metastatic breast cancer (Phase I) NCT02596373. Liposomal mitoxantrone in advanced metastatic breast cancer (Phase II) NCT04791228. ThermoDox + MR-HIFU for relapsed solid tumors (Phase II)	The most mature platform overall, backed by established manufacturing processes and regulatory frameworks, has allowed for the encapsulation of a wide variety of payloads, from classic small-molecule drugs, such as antifungals and chemotherapeutics, to mRNA vaccines. This highlights its versatility and biocompatibility.
**Extracellular Vesicles** **(EVs/exosomes/microvesicles)**	TRL 3–6 (preclinical/early trials)	There are no approved therapies for EVs yet as a regulated commercial product	NCT01779583 Circulating exosomes as predictive biomarkers in advanced gastric cancer NCT02393703. Study of the role of exosome-mediated intercellular signaling in patients with pancreatic cancer NCT01294072. Use of plant exosomes to deliver curcumin to normal tissue and colon cancer (exploratory trial) NCT01668849. Plant exosomes to prevent oral mucositis associated with chemotherapy/radiotherapy in head and neck cancer	It shows great promise due to its biological origin and ability to transport complex molecules. However, it lacks production standards (GMP), robust characterization, and solid clinical data. Clinical translation is still in its early stages.
**Polymersomes (polymer vesicles)**	TRL 2–4 (mainly preclinical)	As far as our knowledge extends, no polymersome-based formulation has achieved approval.	To date, there are few, if any, published clinical trials using polymersomes as a regular therapeutic platform. Their use is mostly limited to preclinical studies.	They offer high versatility: different polymers, the possibility of adapting to stimuli (pH, redox, temperature, etc.), but biocompatibility, biodegradability, and regulatory pathways are not yet fully established. Slow clinical translation.
**Stimuli-responsive/Hybrid/Biomimetic Vesicles** **(hybrid lipid/polymer vesicles)**	TRL 1–3 (concept/in vitro/preclinical)		There are no publicly available reports of major clinical trials. Most developments remain at the research stage.	These platforms combine advanced features (controlled release, stimulus response, targeted delivery, bio-synthetic hybrids), making them conceptually very attractive. However, they face significant challenges: production scale, reproducibility, toxicology, regulation, and a lack of in vivo data.

## Data Availability

No new data were created or analyzed in this study.
